# Remittances, political economy and public health expenditure: evidence from Africa

**DOI:** 10.1093/heapol/czaf089

**Published:** 2025-11-08

**Authors:** Lwanga Elizabeth Nanziri, Judith Kabajulizi, Paul Tema Gbahabo

**Affiliations:** University of Stellenbosch Business School, Belpark Campus, Carl Cronje Drive, Cape Town 7353, South Africa; School of Economics, Finance and Accounting Faculty of Business and Law, Coventry University, Priory Street, Coventry CV1 5FB, United Kingdom; University of Stellenbosch Business School, Belpark Campus, Carl Cronje Drive, Cape Town 7353, South Africa

**Keywords:** migrant remittances, political economy, governance, health expenditure, COVID-19, tax revenue, F20, H2, O23

## Abstract

This article revisits the argument that in the absence of good governance, remittance inflows cause the government to renege on the provision of social services and crowd out public finance where private substitutes exist. Using a quantile approach on a sample of African countries for the period 1990–2022, and after controlling for the endogeneity of remittances, the results show a positive contribution of remittances to public health expenditure, which tis annihilated into a non-linear crowd-out of public health expenditure across quantiles in the presence of varied political regimes. This relationship does not change even in the presence of a health shock. The crowd-out of public health expenditure points to an indirect effect of remittances through household consumption, private investment and tax revenue.

Key messages

- Remittances are a potential source of debt-free budgetary support and could increase fiscal space for health in economies that receive large volumes of migrant remittances.
- Autocratic governance tendencies deprive legislators and the public of the power to negotiate for appropriate budget allocations for the health sector in low-and medium-income countries.
- The remittances effect of a crowding-out of public health expenditure in the presence of a negative health shock suggests that there is minimal room for negotiation by the electorate in the presence of a health crisis.
- Countries that receive large volumes of remittances can strategically leverage remittances to complement their public health budgets, meet electorate demands and work towards meeting the universal health coverage goals.

## Introduction

This study investigates the effect of migrant remittances on public health financing in recipient countries, and the role of governance and a negative health shock in Africa. The World Health Organization (WHO) reports that, in about half of African countries, over 40% of the total health expenditure is constituted of household out-of-pocket payments, with governments’ contribution a meagre 20% as of 2017 global health spending (World Health Organisation 2019). This financing structure is regressive as it contributes to inequality in access to health services. Where governments have attempted to abolish user fees, the resulting public health services provided have been appalling (World Health Organisation 2019). As government contribution towards this sector declines, there has been an observed increase in the contribution of private health financing. Several African governments spend below the 15% budget share agreed upon in the Abuja Declaration of 2001. For instance, between 2000 and 2017, while governments in Africa spent an average of 9% of budget share on healthcare, private health financing amounted to United States Dollars (USD) 768.4 billion, which is 34% higher than the total government expenditure of USD 575 billion over the same period (World Health Organisation 2019). There is emerging evidence that remittances boost private health spending (Ambrosius and Cuecuecha 2013, Yol 2017), but to what extent can they explain the observed public health financing patterns?

During the past decade, the volume of migrant remittances defined as private transfers by migrant residents to their home countries, has increased in recipient countries. For the period 1990–2005, the average remittance flows to developing countries amounted to US$240 billion out of the global total of US$318 billion, and a large proportion went to Africa (Anyanwu and Erhijakpor 2010). By 2014, Algeria, Egypt, Morocco, Nigeria, and Tunisia received 81% of the total flows to Africa. For small African countries like the Comoros, Lesotho and Liberia, remittances represented over 20% of Gross domestic Product (GDP). In 2016, official remittance flows to developing countries reached US$400 million, representing over 70% of the global remittances’ flows. Micro level evidence shows that recipients use remittances mainly for education, healthcare and food consumption (López-Córdova 2005, Zhunio et al. 2012, Adams and Cuecuecha 2013), which would otherwise be paid for through credit. This is because public provision of social services is inadequate in many recipient African countries. But there is a paucity of evidence of the relationship between remittance flows and government decisions on social services budget allocation.

### The role of migrant remittances in domestic healthcare expenditure

Foreign remittances have increased significantly since 2000 and have become a vital source of external financing for many developed and developing countries. Recipients use remittances for immediate expenditure demand such as healthcare, education, and household consumption goods, as well as for private investment (Schoeni 2002, Reil-Heid 2006, Medina and Cardona 2010, Doyle 2015, La and Xu 2017, Amega 2018), which, in turn, reduces any pressures on government to account for public funds when social services delivery is poor. Yet, remittances may affect politically contestable markets, in as far as they reduce the cost of political participation and incentivize individuals to participate in politics. For instance, as repeated receipt of remittances increases, the income and economic security of recipient households reduce their income risk (Doyle 2015), and, potentially, belong to a group of low-cost political participators, capable of removing leaders more easily, so that incumbent politicians will pay close attention to the interests of this group. The State will devote resources to appease this small group by instituting deliberate policies and strategies that ensure a continuous and smooth flow of remittances. An example is the labour externalization policy in Uganda that has seen many unemployed (underemployed) youth exported to the Middle East, mainly as unskilled labour and their contribution rose to 23% of the total remittances in 2018 (Nattabi et al. 2020). In return, this group of low-cost political participators will acquiesce with the incumbent politicians and even lobby for the status quo to remain (Lake and Baum 2001), further reducing the likely pressure on government to increase social expenditure.

Furthermore, where the government claims credit for remittance flows, it may divert resources to areas of low or declining levels of remittances to maintain its popularity. A study on remittances and the crowding-out effect of public finance in Mexico showed that the growth of federal government spending per capita at the municipal level was lowest in areas that received a larger share of private and collective remittances (Collective remittances belonged to a scheme of institutional co-financing of public projects. Mexicans abroad organized in groups, remitted funds for public projects in their areas of origin and the funds were matched by public financing in a three for one ratio. Private remittances are those received by individuals and households.) (Ambrosius 2019). The effect was stronger in municipalities that were strongholds of the ruling party, which had also been in power for seven decades.

Ebeke (2012) also found that remittances reduce public health spending in developing countries with low levels of rule of law, government effectiveness, political stability, and voice and accountability. Although the government is not directly replacing its public health spending obligation with private remittances, as Yol (2017) argued, the resultant substitution of demand for public services with private demand is an incentive for the government not to invest in public services. The increase in demand for private healthcare is particularly so in developing countries where healthcare by private providers is perceived to be of higher quality when compared with public healthcare provision (Brugha and Zwi 1998, World Bank 2011).

Foreign remittances tend to substitute for public spending on social services, particularly in less democratic regimes (Lake and Baum 2001, Ebeke 2012, Easton and Montinola 2017, Mina 2019). As Grabel pointed out, remittances may create a ‘public moral hazard’ for developing country governments (Grabel 2009). By partially resolving bottlenecks, remittances may encourage states in the developing world to ignore their traditional responsibilities because they assume that remittances will fill various voids. Given the autocratic tendencies of many governments in Africa, this paper attempts to investigate the interplay between remittances and social expenditure in some emerging democracies on the continent.

### Political business cycles and healthcare expenditure

Political business cycle approaches (see, e.g. Nordhaus 1975, Rogoff 1990), and the theory of public spending, explain how politics and foreign remittances interact to influence healthcare expenditure policy. The theory of public spending posits that domestic government expenditure is determined by a nexus of state centred and societal centred factors which influence the decisions of policymakers (Swank 1988). For example, in a modern democratic state, party ideology and the pursuit of self-interested actions of elected officials and their bureaucratic appointees, may drive the direction of domestic expenditure and service delivery, a tool often used to enhance election popularity. An evaluation of Organisation for Economic Co-operation and Development (OECD) countries showed that incumbents opportunistically increase public healthcare expenditure in election years, to improve their re-election chances (Potrafke 2010, Herwartz and Theilen 2014), and Leftist governments pursue expansionary expenditure policies (Cusack 1997 , Potrafke 2009). In the economic theory of the state, the provision of services is a function of contestability in political markets (Lake and Baum 2001).

The level of public services delivery depends on the barriers to enter and exit the political market, and the cost of political participation by citizens. Juxtaposing this theory with the Leviathan model of public spending (For the application of Leviathan model in public spending, see Abizadeh and Cyrenne 1997.), which posits that government is a revenue-maximizing monopolist; it implies that the State enjoys a natural advantage in the political market so that the higher the barriers and cost of political participation the more likely are governments to deliver fewer services and earn higher rents. Governments can hold onto power while maximizing rents. On the other hand, governments will deliver more services, and earn lower rents, if the barriers are low and citizens are able to participate in politics at a low cost; features common in democratic systems. Additionally, institutional groups such as businesses, labour unions, and social class-based actors also influence healthcare expenditure policy through efforts to influence the political and economic interests, and policymakers often decide on public expenditure in anticipation of such groups’ actions.

Given the increased remittances flows and the observed household expenditure patterns, this study aims to re-examine the response of governments in remittance-receiving countries. Our study investigates whether ‘remittance flows cause governments in receiving countries to renege on their responsibility to provide social services such as healthcare’. The hypothesis is that as household incomes increase, courtesy of remittances, citizens care less about the dysfunctional public health system and instead increase their demand for private health services. These remittance recipients, who are also the elite voters, do not agitate for better social services (Ambrosius 2019). On the other hand, the very poor, who might not receive supplementary income, have no voice to hold governments accountable to providing the appropriate social services. This makes governments less likely to allocate funds to the public services especially in the rural areas (home to most households in African), where the private sector is less likely to invest in services such as healthcare. This voter-government behaviour has been reported in the literature, e.g. Ahmed (2012), Ebeke (2012), Ambrosius (2019). Strong, adequately financed health systems are essential to ensure both individual and global public health security. This was evidenced by the Ebola crisis in West Africa and by the Coronavirus disease 2019 (COVID-19) pandemic, where demand for healthcare services far outweighed the supply even in rich countries. Although remittances are shown to respond positively to COVID-19 infections in home countries (Kpodar et al. 2023), the multiple effects associated with these health crises suggest that governments may have competing demands for the fiscal coffers in sectors beyond the health sector.

We acknowledge that remittances could potentially influence multiple sectors including education, agriculture, and infrastructure. However, our focus on public health expenditure is supported by both theoretical and empirical evidence. According to the New Economics of Labour Migration framework (Taylor 1999), remittances are often sent to insure against income volatility and health-related risks, especially in contexts where public health systems are underfunded or inaccessible (Grabel 2009). Health shocks require immediate financial responses, making remittances a critical buffer. The magnitude and immediacy of their impact on health are more pronounced when compared with the impact on education or infrastructure investments which tend to have longer gestation periods and are less responsive to short-term household income changes.

Additionally, health services are often more visible and electorally sensitive than infrastructure or agriculture, especially in democratic contexts. Political economy literature suggests that governments in liberal democracies may prioritize health spending due to its visibility, urgency, and electoral salience. For example, using Afrobarometer data spanning 34 African countries, a study found that remittance dependence can influence citizens’ support for taxation and perception of public service delivery (Konte and Ndubuisi 2024). In contexts where remittances reduce reliance on state services, governments may strategically invest in health to reassert relevance and maintain the fiscal contract (García and Maydom 2023).

Our sample comprises of 53 Africa countries, most of whom receive a substantial amount of migrant remittance inflows. While the majority received independence from colonial powers in the early 1960s, they run relatively different political structures. For instance, Tanzania operates a multiparty system with fixed presidential term limits, although one party, whose leadership is heavily contested, has dominated the political arena since independence. On the other hand, Uganda operates a multiparty system, but a single party and leader whose position is ‘not contestable’, has ruled the country, for most of the postindependence era. Kenya’s and Ghana’s experience falls in between the systems in Uganda and Tanzania, with a dynamic entry and exit of political parties. In all these countries; however, the various political parties can field candidates who make up the legislature. Such a political structure should translate into a dynamic composition of incumbents and opposition politicians elected to the legislature which approves sector budgets and performs an oversight role to the budget performance.

Using liberal and electoral democracy as indicators of political participation, we estimate the interactive effect of remittances flows and these democracy indicators on public health expenditure. Given that remittances are endogenous, and the diversity of African countries in terms of growth trajectory and degree of democracy, we employ the smoothed instrumental variable quantile regression technique. Data for this study come from the WHO’s Global Health Expenditure Database, Institute of Health Metrics and Evaluation (IHME), World Bank Worldwide Governance Indicators Project, Variety of Democracy (VDem), and World Development Indicators (WDI) for the period 1990–2022.

The results are three-fold. First, there is evidence of non-linear crowd-out of public health expenditure of up to one percentage points in the presence of strong democracies in the low-medium-income African countries. The results suggest that autocratic tendencies deprive legislators and the public of the power to negotiate for appropriate budget allocations for the health sector in low-and medium-income countries. Second, the crowd-in of public health expenditure is via indirect channels such as an increase in consumption expenditure and private investment for which beneficiaries pay tax such as value-added tax (VAT), with an accompanying crowding-out of private capital investments in the high-income countries. Thirdly, there is no evidence of crowd-in of public health expenditure in the presence of a negative health shock, implying that governments’ expenditure patterns do not change in the presence of a health crisis. Our results are robust to other measures democracy and with the literature. Thus, for countries that receive large volumes of remittances, our results suggest that they can strategically use remittances to complement their public health budgets if they are to meet the universal health coverage goals.

This research contributes to three strands of literature: The first one relates to the relationship between external financing (such as loans, Aid, etc.) and a country's growth and development. Evidence is mixed as some researchers argue that such financing is needed for growth and poverty reduction (Sachs 2005), while others argue that it perpetuates poverty and leads to dependency (Moyo 2010, Easterly 2014). The mechanism is often through the unfavourable repayment terms that burden developing countries, while distorting their economic sectors. However, as remittances become a major component of the external financial inflows, with no debt obligations, there is need for research on the potential of remittance flows to complement Africa’s development efforts such as using them as instruments (such as diaspora bonds) to mobilize resources to finance social services such as healthcare.

The second strand of literature relates to the role of remittances in the political economy. There is evidence that remittances improve the quality of democratic institutions by complementing private investment or crowd out public expenditure (Ambrosius 2019) in autocratic settings. In the latter case, citizens worry more about securing alternative finance to access the services that would otherwise be provided by government. In doing this, they divert their attention from government inefficiencies, and cling onto the stability that allows them to receive their funds from elsewhere. Governments are then re-elected without providing basic services but by piggybacking on the positive sentiments and confidence of citizens who receive remittances. This paper builds on this literature since most African governments have minimal voter participation.

The third strand of literature relates to the role of remittances in domestic resource mobilization efforts. Even though they originate from outside of the domestic economy, governments can benefit from migrant remittances indirectly if the system of VAT exists as an instrument of revenue collection (Ebeke 2010). This happens when recipient households spend on consumption of goods and services in the recipient country. Ebeke (2010) refers to this as the indirect route. In cases where recipients can save and raise the required capital for entrepreneurial activities, they not only graduate to being bankable and thus attract formal finance for their businesses (Giuliano and Ruiz-Arranz 2005), but they can pay income tax if their business activities reach the tax threshold. This would be the direct tax route. However, the indirect route is crucial given that direct tax revenue performs lowest of all tax categories in many developing countries.

## Methodology

### Model specification and data

We are interested in the impact of remittances on public health expenditure. The model specification follows empirical literature on modelling the determinants of public health expenditure in developing economies, specifically focusing on the remittance effect (see Ebeke 2012, Ambrosius 2019). We then introduce an interaction between remittances and. political representation, which is a proxy for the degree of democracy, and an alternative to the worldwide governance index that is often employed in the literature (According to Ebeke (2012), the governance index and its indicators, which are a product of the Worldwide Governance Index Project, are criticized for reflecting perceptions of the citizen rather than the reality on ground.). We assume that in a democratic government, the presence of a reasonable proportion of opposition lawmakers, is a proxy for the voices of the voters. These voters and the lawmakers will agitate for appropriate budget allocations to the public health sector. We therefore consider two variables—‘electoral democracy’ and ‘liberal democracy’. The electoral democracy index is a proxy of the degree of election fairness and freedom of expression in a country We assume that elected representatives are a proxy for the voices of the voters and are likely to agitate for increasing the budget allocations to the health sector in line with population health needs. The index ranges from 0 to 1 with a higher score indicating a more robust electoral democracy, while a lower score suggests limitations or weaknesses in the electoral process. The liberal democracy index captures the extent of a country’s human rights, equality before the law, and legislative and judicial constraints on the executive. The index ranges from 0 to 1 with higher values indicating better governance as in Murshed et al. (2022). These aspects are quite relevant in many African democracies where elections are often marred with unfairness. The addition of such a variable is in line with the view that democracies tend to allocate more funds towards social welfare.

The inclusion of political representation is premised on the argument that remittance inflows constitute a positive income shock, which may increase recipient households’ affordability of private healthcare away from poor quality public healthcare. Since these households are also more likely to be elite voters, Ambrosius (2019) argues that they would be less likely to hold incumbent lawmakers accountable for failure to provide good quality health services.

Our empirical exercise is conducted on African countries for which data is available. The region features a mix of old and recent democracies, which allows us to test our hypothesis that democratic institutions have a role in government budget allocation. African countries also receive a substantial amount of migrant remittances as a percentage of GDP. Finally, Africa is the pioneer of low-cost financial innovations such as mobile money and agent banking, that facilitate swift, safer and user-friendly transfer of funds between migrants and their families (According to remittance price worldwide data for the period 2011–8, Nigeria and Ghana, featured among the largest recipients of diaspora remittances on the Continent.).

Data on public health expenditure, private health expenditure and out-of-pocket payments for health services data are obtained from the WHO’s Global Health Expenditure Database and captured as Domestic General Government Health Expenditure as a percentage of General Government Expenditure (DGGHE_GGE) (Computed as: (transfers from government domestic revenue (allocated to health purposes) + social insurance contributions)/General Government Expenditure (GGE).), domestic private health expenditure as percentage of current health expenditure (DPHE_CHE) and out-of-pocket as percentage of current health expenditure (OOP_CHE), respectively. These databases provide comparable data on a range of public and private health expenditure across countries for the period 2000–23. However, since the scope of this paper is for the period 1990–2022, we had to retrospectively extend the data for the period between 1990 and 2000 using linear extrapolation based on the observed trend of the government health spending (thousands of 2022 United States Dollars) obtained from the Institute for Health Metrics and Evaluation (IHME) Database, which spans from 1995 to 2021. We expand analysis in two ways. First, we replace the dependent variable with domestic private health expenditure as a percentage of current health expenditure (DPHE_CHE). For this variable, we hypothesize that as remittance receipts increase, households’ demand for private healthcare services also increases via both institutional health insurance policy uptake and direct out-of-pocket payments because they can afford it. The result would be an increase DPHE_CHE. We expect a positive and significant relationship if indeed remittances complement private health provision (or crowd out public health finance). Second, we investigate the effect of a health shock such as Ebola epidemic between 2014 and 2018, or the COVID-19 pandemic. We introduce a dummy variable equal to one for the period 2014–22, zero otherwise, to capture both shocks. We hypothesize that these shocks should force governments to increase public health expenditure. This action should also ensue when health aid is abruptly withdrawn, as in the case of the abrupt seizure of the United States Agency for International Development(USAID) funding, or the withdrawal of the USA, which funds almost a fifth of the WHO budget, which sources have supported Africa’s health sector for decades. Thus, we expect an increase in public health expenditure.

Remittances, measured as personal remittances received (current US$), are obtained from the WDI database. This database has a record of economic and development indicators for all countries globally. Additional data on home-destination pairs is obtained from Remittances Prices Worldwide (see www.rpw.org.) database of the World Bank. This pairing is crucial for our identification strategy, as we discuss in the next section.

The degree of democracy is obtained from VDem data as in (Coppedge et al. 2021). For comparability of our results with earlier empirical work, we repeat the empirical work using the traditional World Bank Worldwide Governance Indicators (voice and accountability, political stability, government effectiveness, rule of law, regulatory quality, and control of corruption).

Other covariates are based on the literature. GDP per capita is included to proxy for the size of the economy. We expect growth in GDP per capita to lead to relatively larger expenditure towards social services like healthcare. Conceptually, higher GDP per capita strengthens a government's fiscal capacity by expanding its tax base and revenue potential. A large fiscal capacity allows a government to respond better to public needs and invest in long-term development with potential increase in budget allocations towards social welfare such as education, health, water, and sanitation, etc. Total tax to GDP ratio is used to capture fiscal capacity. We acknowledge that many developing countries have in the past years received budgetary support earmarked to specific sectors such as health, education, water, sanitation, etc. Thus, some empirical work combines grants with taxes, such as Dincecco and Prado (2012), to estimate the impact of external resources. We follow Murshed et al. (2022) and consider all types of taxes for our estimates. We then add official development assistance to the health sector as a percentage of GDP to capture potential inflows that can feed into our sector of interest. The implicit form of our model is specified as follows:


(1) PubHlth=P(REMIT,DEM,REMIT*DEM,X)

In Equation (1), PubHlth represents DGGHE_GGE. In a separate model we replace this variable with domestic private health expenditure to investigate potential substitution between public and private health expenditure. Our interest is the interaction between REMIT (personal remittances received in current US$) and DEM (electoral and liberal democracy) which is our measure of political representation. *X* represents a set of control variables including GDP per capita and fiscal capacity measured by tax revenue as a percentage of GDP. These variables provide a parsimonious model for our estimate (We acknowledge that debt can exert pressure on fiscal capacity, that inflation provides the real value of the public health budget allocation, population growth rate as a proxy for the demand for public healthcare and military expenditure as a discretionary expenditure that can compromise expenditure towards many sectors.).

### Estimation strategy

We are interested in making causal inferences between remittances, public health expenditure and governance. However, there are two sources of potential bias that can compromise our estimates. First is reverse causality. Migration, which is the source of remittances, might have been driven by poor socio-economic conditions (which include poor healthcare) in the migrant's country of origin (Adams and Cuecuecha 2013, Ambrosius and Cuecuecha 2016, Ambrosius 2019). This potential endogeneity requires the use of an instrumental variable. Second is the heterogeneity bias that is associated with the diversity of the countries in the sample such that mean regression estimates (obtained from linear regressions) do not capture the conditional distribution of the estimand (Bitler et al. 2006). This requires a non-linear regression approach. We therefore employ the smoothed instrumental variables quantile regression (SIVQR) approach as in Adams and Cuecuecha (2013), Chernozhukov and Hansen (2013), Ambrosius and Cuecuecha (2016), Kaplan and Sun (2017), Ambrosius (2019).

The SIVQR offers several advantages over the traditional instrumental variable-quantile regression framework, which involves the non-smooth indicator function rather than the much more efficient smoothed estimating equation. Some of these advantages include the following: (i) smoothed estimating equation can yield lower asymptotic mean squared errors in econometric function leading to more accurate estimates. (ii) Smoothing the estimating equations establishes high-order properties of the estimator by exhibiting high-order refinement of the bootstrap replication (Kaplan and Sun 2017). (iii) Smoothing improves computation time and statistical accuracy by utilizing higher bandwidths, i.e. the degree of smoothing applied to the data. Bandwidths control how much weight should be given to each observation when estimating the conditional quantile function.

**Figure 1.**
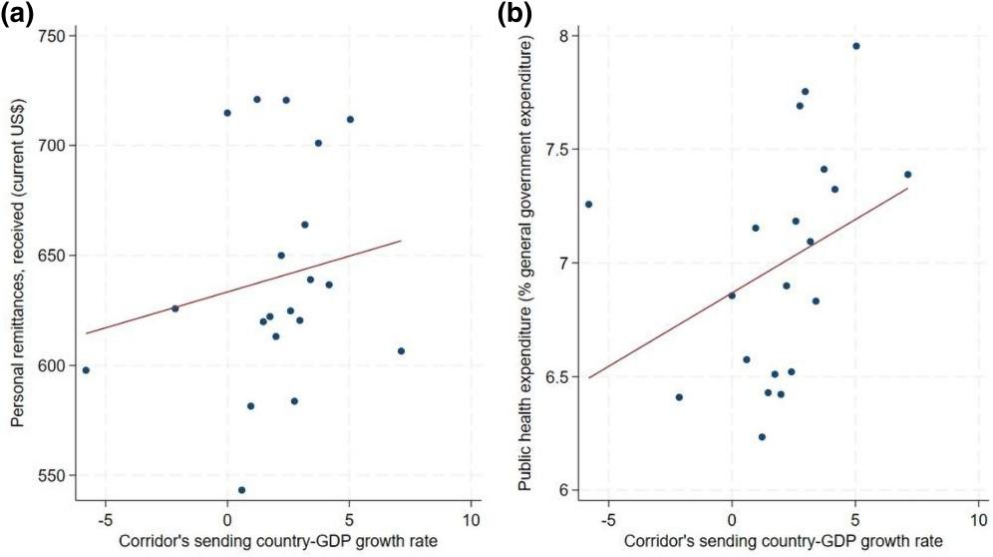
Partial correlations (host country GDP growth rate and remittances) and host country GDP growth rate and public health.

Following Ebeke (2012), we exploit the GDP growth rate of the host country as the instrumental variable of choice. We obtain data from the World Bank's Remittance Prices Worldwide database to determine the remittance corridor to establish the sending (home) and destination (host) countries (A remittance corridor is a channel through which funds flow from one country to another. A disproportionate sum of global remittance transfers is captured in only a few bilateral channels, usually one-way traffic, indicating corridors flow in one direction. Home country refers to the country that sends migrants while host country refers to the destination country of the migrants.). Where there is more than one corridor to a particular country, we take a simple weighted average of the GDP growth. We argue that although the business cycle in the host country correlates with the volume and amount of migrant remittances [thus fulfilling the relevance assumption of instrumental variable analysis: Cov ($Z_{i}X_{i}$) ≠ 0], it does not directly affect budgetary allocation in home countries [thus fulfilling the exogeneity assumption of instrumental variable analysis: Cov ($Z_{i}U_{i}$) =0]. Figure 1a presents the partial correlation between the host country GDP growth rate and migrant remittances received, adjusting for country-fixed effects of selected African economies. In contrast, Fig. 1ba depicts the relationship between public health expenditure and host country GDP.

The estimation follows a two-step approach wherein the first step, remittances received, is regressed on the exogenous corridor's sending-country (host) GDP growth rate, as shown in Fig. 1a and equation (2). The predicted values of remittances are then used as a regressor in the model for public health expenditure in the second stage regression, as represented in equation (3).


(2) $$\mathrm{REMI}T_{t}=\alpha_{1}+\alpha_{2}\mathrm{dHOS}T_{j}+\gamma_{i}+\varepsilon_{t}$$

(3) $$\begin{array}{rl} \mathrm{PubHlt}h_{t}= & \alpha_{3}+\alpha_{4}\hat{\mathrm{REMI}T_{t}}+\alpha_{5}\mathrm{DE}M_{t}+\alpha_{6}\mathrm{DE}M_{t}*\hat{\mathrm{REMI}T_{t}}\\ & +\beta X_{t}+\gamma_{i}+\varepsilon_{t} \end{array}$$

where dHOST captures the GDP growth in the host country, $\hat{\mathrm{REMI}T_{t}}$ is the predicted value of remittances from the first stage regression, $\gamma_{i}$ captures the receiving (home) country's fixed effects and $\varepsilon_{t}$ is the idiosyncratic error term. Equation (3) provides our main results and is estimated using the SIVQR (Kaplan and Sun 2017). Figure 1a shows that our IV is relatively more correlated with migrant remittances compared with the correlation with our dependent variable (public health expenditure) in Fig. 1b, a key condition in validating our instrument.

### Descriptive statistics

From the statistics in Tables 1 and 2, there is no explicit pattern between remittance flows and the public health expenditure in the countries in various quantiles. Similarly, the statistics are mixed regarding the amount of remittances received by low, medium and high-income countries. for instance, some of the highest recipients of remittances (Carbo Verde, Eswatini, South Africa, Tunisia) also spend a reasonable percentage of their budget on public health, while other high remittance recipients like Egypt and Lesotho spend relatively less. On the other hand, the relationship between public health expenditure and regime type is even more nuanced. For instance, Botswana and Carbo Verde receive high remittances, spend on public health and they feature democratic regimes, whereas Eswatini receives high remittances, spends on public health but features an autocratic regime. Paradoxically, Chad, which receive low remittances, spend relatively high percentage of its budget on public health and it features an autocratic regime. The sample is however, split between autocratic and democratic regimes (27 and 26 countries, respectively).

**Table 1.** Mean statistics of remittances, public health, electoral democracy index, electoral democracy index, governance index, and political regime type for selected African countries.

Economies in the sample	Remittances inflows (USD millions)	Public health (% general govt expenditure)	Electoral democracy	Liberal democracy	Worldwide governance index	Regine type
Upper quantile						
Botswana	810	8.16	0.69	0.59	0.60	Democracy
Equatorial Guinea	87	2.16	0.18	0.05	−1.42	Autocracy
Gabon	636	6.04	0.35	0.21	−0.68	Autocracy
Mauritius	682	7.59	0.71	0.61	0.70	Democracy
Namibia	756	13.15	0.65	0.53	0.27	Democracy
Seychelles	615	8.48	0.45	0.36	0.40	Democracy
South Africa	889	13.09	0.67	0.56	0.30	Democracy
Libya	134	6.09	0.15	0.09	−1.43	Autocracy
Median quantile						
Algeria	486	7.70	0.30	0.15	−0.80	Autocracy
Angola	295	4.29	0.21	0.10	−1.16	Autocracy
Benin	688	5.73	0.58	0.47	−0.37	Democracy
Cabo Verde	646	9.28	0.76	0.67	0.43	Democracy
Cameroon	550	3.73	0.30	0.15	−1.02	Autocracy
Comoros	374	4.59	0.39	0.20	−1.05	Democracy
Congo	611	3.31	0.26	0.13	−1.55	Autocracy
Côte d'Ivoire	534	4.69	.	.	−1.20	Democracy
Djibouti	568	5.62	0.24	0.11	−0.87	Autocracy
Egypt	725	4.90	0.20	0.14	−0.59	Autocracy
Eswatini	936	10.08	0.13	0.10	−0.61	Autocracy
Ghana	651	7.58	0.64	0.54	−0.02	Democracy
Guinea	726	2.79	0.26	0.11	−1.09	Autocracy
Kenya	745	7.25	0.43	0.31	−0.58	Democracy
Lesotho	881	8.18	0.49	0.38	−0.27	Democracy
Mauritania	301	3.83	0.35	0.16	−0.71	Autocracy
Morocco	808	5.21	0.24	0.22	−0.29	Autocracy
Nigeria	525	3.63	0.40	0.27	−0.99	Democracy
Sao Tome and Pri	536	11.68	0.65	0.53	−0.35	Democracy
Senegal	501	6.77	0.67	0.51	−0.16	Democracy
Tanzania	631	8.32	0.70	0.58	−0.49	Democracy
Tunisia	681	10.80	0.36	0.26	−0.13	Autocracy
Zambia	540	7.29	0.45	0.37	−0.53	Democracy
Zimbabwe	299	9.17	0.28	0.19	−1.38	Autocracy
Lower quantile						
Burkina Faso	889	6.30	0.51	0.33	−0.40	Democracy
Burundi	435	5.53	0.23	0.12	−1.21	Autocracy
Central African	65	6.62	0.33	0.19	−1.32	Autocracy
Chad	75	7.96	0.25	0.09	−1.31	Autocracy
Democratic Republic of Congo (DRC)	294	2.85	0.26	0.10	−0.81	Autocracy
Eritrea	67	2.21	0.08	0.02	−1.45	Autocracy
Ethiopia	756	6.25	0.24	0.11	−0.87	Autocracy
Gambia	434	6.13	0.32	0.20	−0.61	Autocracy
Guinea-Bissau	628	2.70	0.39	0.23	−1.18	Democracy
Liberia	543	3.97	0.45	0.32	−1.02	Democracy
Madagascar	573	11.18	0.43	0.23	−0.60	Democracy
Malawi	687	6.46	0.47	0.38	−0.46	Democracy
Mali	776	5.27	0.51	0.34	−0.56	Democracy
Mozambique	757	5.66	0.40	0.27	−0.58	Democracy
Niger	660	7.90	0.50	0.37	−0.65	Democracy
Rwanda	573	6.38	0.19	0.13	−0.46	Autocracy
Sierra Leone	802	5.41	0.41	0.25	−0.88	Democracy
Somalia	52	4.51	0.17	0.07	−2.02	Autocracy
Sudan	766	9.63	0.17	0.05	−1.43	Autocracy
Togo	665	3.91	0.36	0.20	−0.92	Autocracy
Uganda	545	6.66	0.30	0.23	−0.58	Autocracy

Source: Authors’ compilation.

The regime type is obtained by taking the mean of the electoral index, and assigning a value of zero to all countries below the mean, and a value of one to the rest of the countries.

**Table 2.** Descriptive statistics for the full sample.

Variable	Obs.	Mean	Std. dev.	Min	Max
Remittances (millions of US$)	1749	877 029 587.73	453.35	0.00	31 487 000 000.00
Domestic general government health expenditure (% general government expenditure)	1739	6.51	3.39	8.42	32.40
Tax revenue (% of gdp)	1486	13.80	324.01	0.60	60.95
Official development assistance in health sector	1413	155 428.58	1.67	0.00	1 686 488.00
External debt (% of GDP)	1716	74.88	486.88	2.32	1343.27
Household consumption per capita (US$ Millions)	1749	1373.11	419.51	119.65	13 789.27
Out of pocket health expenditure (% of total household consumption expenditure)	1185	40.26	20.20	3.00	86.50
Electoral democracy	1716	0.39	0.20	0.07	0.82
Liberal democracy	1716	0.27	0.19	0.01	0.72
Governance	1272	−0.69	0.60	−2.24	0.91
Corridor GDP growth rate	1749	2.14	2.65	−14.55	12.00
Domestic private health expenditure (% current health expenditure)	1185	47.17	18.49	7.50	89.50
Health shock (Ebola and COVID-19)	1749	0.27	0.45	0.00	1.00

Source: Authors’ compilation.

The summary statistics in Table 2 show that the countries in our sample spend on average 6.5% of their budget on public health, which is less than the target set by the Abuja Declaration of 2001. Out-of-pocket health expenditure averages 40%, in line with the reports by the WHO. However, tax revenue at 13%, is comparable to the 2021 global average of 17.1%.

## Results and discussion

We are interested in the moderating effect of democracy (which is the measure of political participation in this study) on the potential negative effect of remittances flows on the budget allocation towards public health expenditure. We hypothesize that in the absence of the opposing voices of the elites, amidst household level remittances, governments will renege on their responsibility to finance social services like public health. In Table 3, we report the results of a baseline model of the determinants of public health expenditure. We then introduce the measures of democracy (electoral and liberal democracy) in Table 4, focussing on the interaction between remittances and these variables.

**Table 3.** Smoothed IV quantile regression estimates of the effect of remittance inflows on domestic public health expenditure.

	Pub health	Pub health	Pub health	Private health	Private health	Private health
Variables	0.25	0.5	0.75	0.25	0.5	0.75
Remittances	0.0020*** (0.0007)	0.0006 (0.0010)	−0.0007 (0.0005)	0.0015 (0.0010)	0.0022 (0.0015)	0.0018 (0.0012)
Tax (% of GDP)	0.0008* (0.0004)	0.0016*** (0.0004)	0.0033*** (0.0006)	−0.0001 (0.0006)	−0.0004 (0.0006)	0.0017 (0.0013)
Health aid	0.3657*** (0.0838)	0.5770*** (0.0847)	0.7960*** (0.0534)	0.2165** (0.1058)	0.5928*** (0.1252)	1.3972*** (0.1119)
External debt (% of GDP)	0.0009*** (0.0003)	0.0010*** (0.0003)	0.0013*** (0.0003)	−0.0008** (0.0004)	−0.0014*** (0.0005)	−0.0030*** (0.0006)
Household consumption per capita	0.0001 (0.0003)	0.0005 (0.0003)	0.0008* (0.0004)	−0.0004 (0.0004)	0.0011** (0.0005)	0.0007 (0.0010)
Out-of-pocket payments	−0.0515*** (0.0065)	−0.0572*** (0.0070)	−0.0702*** (0.0084)	0.9913*** (0.0083)	0.9439*** (0.0111)	0.8570*** (0.0175)
bwidth_req	0.84	0.83	0.82	1.38	1.30	1.62
bwidth_max	1.71	4.50	1.57	4.19	7.06	3.09
Observations	868	868	868	868	868	868

Robust standard errors in parentheses, ***P < .01, **P < .05, *P < .1.

The smoothing bandwidth (bwidth_req) used in SIVQR denotes the degree of smoothing applied to the data. It is a parameter that controls how much weight should be given to each observation when estimating the conditional quantile function. (bwidth) < (bwidth_max) is desirable otherwise it would be indicative of a weak instrument. Using the ‘ivregress estat firststage’ test for a weak instrument, the null hypothesis was rejected. Instruments used: host country GDP growth rate.

**Table 4.** SIVQR estimates of the interaction between remittances and public health expenditure.

	(1)	(2)	(3)	(4)	(5)	(6)
Variables	0.25	0.25	0.5	0.5	0.75	0.75
Remittances*electoral democracy	−0.0166*** (0.0035)		−0.0226*** (0.0020)		−0.0291*** (0.0019)	
Remit*liberal democracy		−0.0194*** (0.0063)		−0.0264*** (0.0027)		−0.0343*** (0.0022)
Tax (% of GDP)	0.0011** (0.0005)	0.0013** (0.0006)	0.0016*** (0.0003)	0.0016*** (0.0003)	0.0020*** (0.0006)	0.0019*** (0.0006)
Health aid	−0.1296 (0.0977)	−0.0263 (0.1069)	−0.1489** (0.0613)	0.0113 (0.0568)	−0.1113 (0.1039)	0.0800 (0.0961)
External debt (%of GDP)	0.0008** (0.0003)	0.0010*** (0.0004)	0.0010*** (0.0003)	0.0012*** (0.0003)	0.0007*** (0.0003)	0.0011*** (0.0003)
GDP per capita	−0.0008**	−0.0008*	−0.0011***	−0.0011***	−0.0011***	−0.0012***
	(0.0004)	(0.0004)	(0.0003)	(0.0003)	(0.0003)	(0.0003)
Remittances	0.0088*** (0.0012)	0.0074*** (0.0013)	0.0114*** (0.0010)	0.0093*** (0.0011)	0.0139*** (0.0013)	0.0112*** (0.0012)
Out of pocket	−0.0692*** (0.0085)	−0.0570*** (0.0086)	−0.0774*** (0.0073)	−0.0667*** (0.0071)	−0.0917*** (0.0071)	−0.0814*** (0.0069)
Electoral democracy (edem)	17.3579*** (3.2868)		22.1710*** (1.3847)		27.2322*** (1.5498)	
Liberal democracy (libdem)		19.0648*** (5.6094)		24.8248*** (1.6150)		31.5345*** (1.7367)
bwidth_req	0.68	1.00	0.69	0.69	0.76	0.78
bwidth_max	1.62	2.46	4.15	4.15	1.56	1.65
Observations	849	849	849	849	849	849

Robust standard errors in parentheses, ***P < .01, **P < .05, *P < .1.

The results in Table 3 Column (1) show that remittances significantly increase public health expenditure in countries in the 25th quantile only. On the other hand, across all quantiles (Columns 2 and 3), an increase in tax to GDP ratio, health aid and external debt to GDP, lead to more funds allocated to towards the health sector (Although a negative effect of external debt is expected (such as Coccia and Benati 2024), the positive result might reflect debt targeted to the health sector either conditionally or due to government priority.). As expected, an increase in out-of-pocket health expenditure, leads to a decrease in the allocation of funds to the health sector. In Columns 4–6, we examine the possibility of crowding in of private health expenditure driven by the remittance. However, the results do not support this hypothesis in the baseline model. Moreover, an increase in health aid and out-of-pocket expenditure leads to an increase in private health expenditure across all quantiles.

The interaction terms in Table 4 Columns (1)–(6) confirm our hypothesis that the positive effect of remittances on public health expenditure can be attenuated by the presence of a non-democratic political representation. We see a significant decline of between one to three percentage points in public health expenditure across all income quantiles, irrespective of the measure of democracy. The results also show that a unit increase in remittances alone leads to an increase in public health expenditure across all quantiles. A similar positive and significant effect is observed for democracy. In both instances the effect is linear across countries. Notice that the effect of the individual variables is positive and significant, while the interactive effect is negative and significant. This implies that the relationship between these variables is not straightforward (Aiken and West 1991, Cohen et al. 2003, Dawson 2014), that is, as the effect of remittances increases, the positive effect of democracy diminishes. This complexity informs our next exercise to split the sample of countries by the degree of democracy. These results are reported in Table 5. The effect of other control variables mimics the baseline results, where an increase in tax revenue leads to an increase in public health expenditure, and an increase in out-of-pocket expenditure leads to a decline in public health expenditure. In this model the effect of health aid is to significantly reduce public health expenditure for countries in the 75th quantile, but no significant effect in the rest of the quantiles, while external debt has a positive and significant effect across all quantiles. We do not pursue the case for private health expenditure as our results showed no statistically significant effect in the baseline model.

**Table 5.** SIVQR estimates of the effect of remittances on public health under different political regime.

	(1)	(2)	(3)	(4)	(5)	(6)
	Autocratic	Democratic	Autocratic	Demomocratic	Autocratic	Democratic
Variables	0.25	0.25	0.5	0.5	0.75	0.75
Log remittances	0.0039*** (0.0014)	−0.0036 (0.0113)	0.0031** (0.0012)	−0.0065*** (0.0023)	0.0049*** (0.0014)	−0.0092*** (0.0009)
Tax (% of GDP)	0.0011 (0.0012)	−0.0001 (0.0027)	0.0015** (0.0006)	0.0015* (0.0008)	0.0022* (0.0011)	0.0020* (0.0011)
Log health aid	0.0994 (0.1404)	0.7175 (0.9240)	0.3878*** (0.1483)	1.1691*** (0.1808)	0.5409*** (0.1970)	1.5847*** (0.0853)
External debt (%of GDP)	0.0026*** (0.0006)	0.0009 (0.0017)	0.0019*** (0.0006)	0.0006 (0.0005)	0.0013* (0.0007)	0.0015*** (0.0005)
GDP per capita	−0.0007 (0.0014)	0.0000 (0.0004)	−0.0011* (0.0006)	−0.0004 (0.0006)	−0.0019** (0.0008)	0.0000 (0.0005)
Out of pocket	−0.0474*** (0.0121)	−0.0300 (0.0321)	−0.0578*** (0.0120)	−0.0529*** (0.0154)	−0.0665*** (0.0125)	−0.0797*** (0.0134)
bwidth_req	0.81	1.00	0.81	0.76	1.03	1.29
bwidth_max	2.23	2.46	4.64	4.69	6.01	3.69
Observations	354	514	354	514	354	514

Robust standard errors in parentheses, ***P < .01, **P < .05, *P < .1.

The results in Table 5 are obtained by first splitting the sample into countries that have a below-the-mean score in electoral democracy, considered to have autocratic regimes, while the rest of the countries are considered to have democratic regimes. Given that the indicators of democracy variables yielded similar results in Table 4, the split was based on the electoral democracy variable. Note that even with this classification, we retain the quantiles generated from the countries’ income category, which provides two layers of heterogeneity. The results show that remittances lead to an increase in public health expenditure in autocratic regimes, but the relationship is non-linear as shown in Columns (1), (3), and (5). In the 25th and 75th quantiles, remittances lead to an increase of four and five percentage points respectively, at a 99% level of significance, while at the median the increase is three percentage points at a 95% level of significance. In the democratic regimes, remittances lead to a decline in health expenditure of up to nine percentage points in middle-high income countries only [Columns (4) and (6)], and the relationship is linear for these countries. Thus, the results in Table 5 support a non-linear relationship between remittances and public health expenditure as we envisaged, resulting from the political and economic diversity of the African countries. This non-linearity is the first contribution of this study, and these results are the main findings of the political economy of remittances on public health expenditure. We therefore argue that the reneging behaviour by governments, found by earlier empirical work as remittance flows increase (Ebeke 2012, Ambrosius 2019), is in medium-to-high income countries with democratic regimes. Therefore, the effect cannot be generalized even in a sample of developing countries because of the complex political and economic heterogeneity.

The non-linearity relationship in autocratic regimes implies that remittances can act as a substitute or complement to public spending. Remittances can substitute for public services, especially when governments underprovide health services, but they can also serve as a complement, prompting governments to increase spending to maintain legitimacy or respond to public pressure. This suggests that the effect depends on the volume of remittances and the political incentives of the regime. At low levels, remittances may reduce pressure on the government, leading to less public investment. At moderate levels, they may increase expectations, prompting governments to invest more. At high levels, governments may again withdraw, assuming households can self-finance health needs. This behaviour aligns with theories of political economy and authoritarian responsiveness, where regimes selectively respond to citizen needs to maintain control without democratizing. Unlike democracies, where public spending is often tied to electoral accountability, autocracies may adjust spending based on strategic calculations—e.g. increasing health expenditure in response to remittance inflows to prevent unrest or co-opt support.

The non-linearity relationship result of our study resonates with Mora-Rivera et al. (2024) who analysed Mexican regions and found that international remittances significantly boost health spending, especially in underserved areas but the effects differ by region and remittance type, reinforcing context-specific non-linearity. Similarly, Imam et al. (2024) showed that remittance flows respond to domestic uncertainty, and in countries with low public spending, remittances increase as a social safety net, reinforcing the idea of non-linear and context-dependent effects. This non-linearity finding has the following implications for policy. First, because the impact of remittances changes at different levels, policies must be tailored to the specific context. For example, countries receiving small remittance inflows may need different strategies than those receiving large amounts. Second, when remittances rise, it may reflect greater expectations from citizens for better services and autocratic regimes might respond by increasing health spending to maintain legitimacy. Policymakers can use remittance trends as a proxy for public pressure and adjust spending accordingly. Third, since the relationship is non-linear, budgeting for health should be adaptive, governments can build in mechanisms to scale spending up or down based on remittance trends and their observed effects.

To examine the effect of a negative health shock, (Ebola epidemic and the COVID-19 pandemic), on public health spending decisions, the results in Table A1 show that for the COVID-19 shock, only in the high-income countries does a significant increase in public health ensue, while the effect of both Ebola and COVID-19 was to reduce public health spending in the low-income countries as shown in Column (2) of Table A2. The effect of remittances remains consistent with the main results. The implication is that even in the face of a health crisis, the reneging behaviour of governments towards public health spending may not change, except in high-income countries. Thus, low- and medium-income African countries may not change their public health spending patterns following the recent USAID closure, if remittances flows continue.

### Robustness tests

We check for robustness by re-estimating Model (3) using the World Bank Worldwide Governance Indicators. First, we construct a composite governance index as a simple average of the six indicators as listed earlier. We further re-estimate the model by replacing the composite governance index with the individual governance. We use the standard normal units of these indicators as they are provided in the data. The indices range between −2.5 and 2.5, with higher values reflecting better outcomes.

Results in Table A3 are consistent with the main results. They are reported at the median to compare with earlier empirical work that has concentrated on the average treatment effect such as Ebeke (2012).

### Transmission mechanism

We proceed in three steps for the transmission mechanism. Literature argues that remittances increase the indirect tax revenue such as VAT, which government can then use to finance social services such as healthcare (Ebeke 2010). Empirical results at micro level show that households use remittances for consumption, health, education services or for private investment (Doyle 2015, Ajaero et al. 2018, Nanziri and Mwale 2023). Thus, remittance recipients are likely to pay tax when they make these expenses. First, we examine whether remittances increase general household expenditure as a percentage of GDP or gross private capital formation (Lequiller and Blades 2014). Second, we examine whether these expenses contribute to tax revenue. Third, for the negative result obtained in countries with democratic regimes, we examine whether remittances increase out-of-pocket health expenditure, causing governments to renege on their obligation to fund the health sector. For this third exercise, we use the split sample of autocratic versus democratic regimes. The results are reported in Tables 6–8.

**Table 6.** SIVQR estimates of the effect of remittances on household consumption and private capital investment.

	Household consumption	Household consumption	Household consumption	Private investment	Private investment	Private investment
Variables	0.25	0.5	0.75	0.25	0.5	0.75
Remittances	0.2393* (0.1441)	0.7730*** (0.1181)	2.6321 (2.2294)	−0.0474* (0.0251)	−0.0846** (0.0335)	0.5475*** (0.1266)
Private investment	0.4301*** (0.0942)	0.3072* (0.1609)	0.4725 (0.5868)			
Tax (% of GDP)	0.1125 (0.0860)	0.1458*** (0.0506)	0.6007 (1.2023)	−0.0033 (0.0108)	0.0140 (0.0158)	0.0347 (0.0618)
Health aid	0.0000 (0.0001)	−0.0002*** (0.0001)	−0.0002 (0.0008)	0.0000 (0.0000)	0.0002*** (0.0000)	0.0003*** (0.0001)
External debt (% of GDP)	0.0720* (0.0432)	0.1495*** (0.0472)	0.2377 (0.6904)	−0.0067 (0.0089)	−0.0161 (0.0141)	−0.0119 (0.0231)
Electoral democracy	41.0879 (70.9337)	−51.6860 (42.9997)	−297.3895 (607.9588)	5.2745 (9.9743)	4.5612 (14.5396)	−99.2293** (39.1731)
Income levels	−40.5961 (30.8825)	−17.4766 (31.7321)	290.0757 (464.4683)	7.9215* (4.5286)	32.2525*** (7.7993)	13.2147 (19.7667)
Household consumption per capita				0.0076 (0.0068)	0.0333*** (0.0119)	0.0486 (0.0408)
bwidth_req	115.84	140.50	471.42	65.16	64.51	89.90
bwidth_max	1008.55	771.53	1550.55	142.21	354.26	173.27
Observations	1198	1198	1198	1198	1198	1198

Robust standard errors in parentheses, ***P < .01, **P < .05, *P < .1.

**Table 7.** SIVQR estimates of the effect of household consumption and private investments on tax revenue.

	Tax ratio	Tax ratio	Tax ratio	Tax ratio	Tax ratio	Tax ratio
Variables	0.25	0.5	0.75	0.25	0.5	0.75
Household consumption	0.2274 (0.1801)	0.6131*** (0.0940)	1.5107*** (0.0944)			
Private investment				0.1829 (1.4462)	1.0202 (2.9910)	3.0764*** (0.1382)
Remittances	0.0029 (0.0523)	0.0305 (0.0328)	−0.0460 (0.0673)			
Health aid	−0.0001 (0.0001)	−0.0001* (0.0001)	−0.0003** (0.0001)	−0.0002 (0.0006)	−0.0002*** (0.0001)	−0.0003*** (0.0001)
External debt (% of GDP)	0.0088 (0.0418)	−0.0543 (0.0338)	−0.1545*** (0.0529)	0.0292 (0.0364)	0.0084 (0.1034)	0.1991*** (0.0552)
Electoral democracy	−128.8019*** (48.0559)	−127.4925*** (31.2383)	−174.1517** (69.4323)	−126.1811 (129.1015)	−106.1601 (104.8152)	−68.5339 (77.5720)
Income levels	38.2079 (46.4354)	99.3562*** (20.8547)	157.2747*** (30.5397)	8.1655 (90.3621)	105.1090 (69.4471)	177.2839*** (36.2488)
GDP per capita				0.1250*** (0.0361)	0.1329 (0.1006)	0.2263*** (0.0502)
bwidth_req	84.73	105.48	105.48	82.52	102.46	212.63
bwidth_max	313.26	548.58	646.77	506.76	534.45	389.28
Observations	1225	1225	1225	1198	1198	1198

Robust standard errors in parentheses, ***P < .01, **P < .05, *P < .1.

**Table 8.** SIVQR estimates of emittances and out of pocket health expenditure in the presence of political regimes.

	Out-of-pocket	Out-of-pocket	Out-of-pocket	Out-of-pocket	Out-of-pocket	Out-of-pocket
	Auto	Demo	Auto	Demo	Auto	Demo
Variables	0.25	0.25	0.5	0.5	0.75	0.75
Remittances (ln)	0.0364*** (0.0073)	0.0319 (0.0425)	0.0288*** (0.0075)	0.0203 (0.0195)	0.0110 (0.0087)	−0.0095** (0.0047)
Tax (% of GDP)	0.0129** (0.0059)	0.0096 (0.0122)	0.0083** (0.0040)	0.0148** (0.0064)	0.0064 (0.0057)	0.0054 (0.0048)
Public health	−2.2035*** (0.3660)	−2.5265*** (0.9669)	−2.5545*** (0.4180)	−2.5958*** (0.4582)	−3.4754*** (0.5664)	−2.6960*** (0.3024)
Health aid	0.6641 (0.7693)	0.5192 (2.6472)	2.5592** (1.2123)	1.7988 (1.6085)	5.2967*** (1.4169)	5.2632*** (0.5393)
External debt (% of GDP)	0.0114*** (0.0032)	0.0055 (0.0065)	0.0112*** (0.0040)	0.0090*** (0.0028)	0.0134*** (0.0042)	0.0119*** (0.0021)
GDP per capita	−0.0133** (0.0057)	0.0005 (0.0027)	−0.0137*** (0.0044)	0.0054 (0.0033)	−0.0061 (0.0053)	0.0064** (0.0027)
bwidth_req	6.24	2.23	5.53	6.19	6.48	6.41
bwidth_max	13.44	19.26	33.96	36.15	21.62	18.42
Observations	354	514	354	514	354	514

Robust standard errors in parentheses, ***P < .01, **P < .05, *P < .1.

The results in Table 6 show that remittances increase consumption expenditure in low-to-middle income countries [Columns (1) and (2)], and they crowd-out private capital investment, but they increase private investment in the upper quantiles, akin to studies in developed countries (Schoeni 2002, Reil-Heid 2006, La and Xu 2017). The first result resonates with the pessimist’s view that remittances drive dependency of recipients (Kpodar and Goff 2011), who forego work, let alone make capital investments that can provide future income streams. Reconciling this last finding with Nanziri and Mwale (2023), the types of investments that households make from remittances are most likely to be micro businesses that support short-term consumption. Another explanation is that in the lower quantiles, households’/countries’ immediate needs are consumption related as opposed to capital investments like construction. For relatively wealthy countries in the upper quantiles, which can afford to support the migration process of household members, the funds that they receive are more likely to go towards capital investment (Reference can be made to the classification of countries provided in Table 1.).

A second set of results in Table 7 is a positive and significant effect of household consumption and private investment on tax ratio but only in upper quantiles (Columns 2, 3, and 6). The lack of significance in the 25th quantile may imply that expenditure is on tax free goods and services such as food from the market, while private investment in the 25th and 50th quantiles might be in informal or non-registered businesses. This result seems to corroborate Ebeke (2010)’s argument that remittances contribute to indirect taxes. The out-of-pocket results in Table 8 show a positive and significant effect of remittances only in autocratic regimes in low-middle income African countries (Columns 1 and 2), but negative and significant in the high-income countries with democratic regimes (Column 6). We can therefore argue that the contribution of remittances to public health that is observed in Table 3, is through tax revenue obtained from household consumption items. It is this revenue that government uses to allocate funds to the health sector as suggested by Ebeke (2010). The direct contribution of remittances reported in Table 5 may refer to direct tax charged on remittances themselves. This practice has been observed in some African countries such as Uganda where a withdrawal charge on remittances is imposed and goes directly to the government, especially targeting remittances received through the mobile money transfer platform. However, this practice may not be an ideal domestic resource mobilization strategy if recipients opt for informal mechanisms such as using friends or public transport as avenues to transfer funds to the beneficiaries. On the other hand, countries could adopt the Mexico model where migration is institutionalized through the existence of home and host country agencies. The host agency receives migrant wages and remits a portion to the home agency. The latter disburses the funds for projects (such as building healthcare centres) that have been agreed upon under a co-funding arrangement with local governments (see Ambrosius 2019). Other mechanisms where diaspora have demonstrated critical resilience capabilities include the Sierra Leone UK Diaspora Ebola Response Taskforce during the 2014–6 Ebola outbreak, the Sudanese American Physicians Association which supports hospitals and health centres in Sudan, and telemedicine services and medical camps such as Telekyanmar in Myanmar during COVID-19 (Dafallah et al. 2025).

## Conclusion

This study examines the interactive effect of remittances and political participation on budgetary allocation towards public services such as healthcare. We revisit a tested hypothesis that remittance flows cause governments to renege on their responsibility to provide public services when democracy is lacking. We use a sample of 53 African countries over the period 1990–2022, control for sample heterogeneity and for potential endogeneity of remittances. The results show a non-linear (negative/positive) relationship between remittances, political participation and public health expenditure. The observed positive effect of remittances on public health expenditure is via an indirect route—through increased household consumption, from which government earns tax revenue and then uses this increased fiscal potential to increase the budgetary allocation to the health sector. The results also show that the presence of a negative health shock does necessarily change governments’ public health spending patterns except in high-income countries. We also find evidence of private capital formation in high-income countries.

The article shows the potential for remittances to contribute to health sector financing. However, the non-linear effect implies that countries will use different strategies based on remittances inflows. Moreover, remittance trends can act as a proxy for public pressure, where a rise in remittances may provide autocratic regimes with an opportunity to increasing health spending and meet citizens’ expectations for better services, thus maintaining legitimacy. Finally, since the relationship is non-linear, budgeting for health should be adaptive, governments can build in mechanisms to scale spending up or down based on remittance trends and their observed effects.

## Data Availability

Data will be made available on request.

## Reflexivity statement

The authors are two females and one male and span multiple levels of seniority. While the first author specializes in development financing modalities in emerging and developing economies, the second author specializes in health economics, and the third author specializes in modelling. All authors have extensive experience in conducting quantitative research.

## Appendix

**Table A1.** SIVQR estimates of remittances, democracy and public health expenditure, controlling for a health shock (COVID-19).

	Public health	Public health	Public health	Public health	Public health	Public health
Variables	0.25	0.25	0.5	0.5	0.75	0.75
Log remittances	0.0021*** (0.0007)	0.0097*** (0.0012)	0.0007 (0.0010)	0.0133*** (0.0012)	−0.0006 (0.0005)	0.0148*** (0.0013)
Remittances*electoral democracy		−0.0191*** (0.0035)		−0.0272*** (0.0023)		−0.0313*** (0.0020)
Electoral democracy		18.5842*** (3.2540)		25.0407*** (1.6444)		28.7594*** (1.6364)
Tax (% of GDP)	0.0008* (0.0004)	0.0008 (0.0005)	0.0016*** (0.0004)	0.0011*** (0.0004)	0.0033*** (0.0006)	0.0020*** (0.0006)
Official Development Assistance (ODA) in Health (ln)	0.3643*** (0.0836)	−0.2411** (0.1041)	0.5746*** (0.0826)	−0.3191*** (0.0726)	0.8002*** (0.0536)	−0.2487** (0.1026)
External debt (% of GDP)	0.0010*** (0.0003)	0.0008** (0.0003)	0.0011*** (0.0003)	0.0009*** (0.0003)	0.0013*** (0.0003)	0.0010*** (0.0003)
Household consumption per capita	0.0002 (0.0003)	0.0005* (0.0003)	0.0005* (0.0003)	0.0007** (0.0003)	0.0008* (0.0004)	0.0005 (0.0004)
Out-of-pocket payments	−0.0524*** (0.0067)	−0.0703*** (0.0087)	−0.0595*** (0.0069)	−0.0845*** (0.0073)	−0.0720*** (0.0085)	−0.1007*** (0.0079)
COVID-19	−0.4983 (0.4810)	0.2239 (0.5235)	−0.6703 (0.4196)	0.2007 (0.3979)	−0.6821 (0.4961)	0.8952* (0.4736)
bwidth_req	0.84	0.71	0.83	0.76	0.82	0.84
bwidth_max	1.81	1.75	4.74	4.75	1.65	1.85
Observations	868	849	868	849	868	849

Robust standard errors in parentheses, ****P* < .01, ***P* < .05, **P* < .1.

**Table A2.** SIVQR estimates of remittances, democracy and public health expenditure controlling for health shocks (Ebola and COVID-19).

	(1)	(2)	(3)	(4)	(5)	(6)
Variables	0.25	0.25	0.5	0.5	0.75	0.75
Remittances	0.0021*** (0.0007)	0.0089*** (0.0011)	0.0011 (0.0011)	0.0132*** (0.0012)	−0.0001 (0.0006)	0.0145*** (0.0014)
Remittances*electoral democracy		−0.0167*** (0.0032)		−0.0269*** (0.0023)		−0.0303*** (0.0021)
Electoral democracy		16.5721*** (2.9177)		24.8176*** (1.5936)		28.0110*** (1.6566)
Tax (% of GDP)	0.0004 (0.0004)	0.0006 (0.0004)	0.0015*** (0.0004)	0.0010*** (0.0004)	0.0031*** (0.0006)	0.0020*** (0.0006)
Health aid	0.4110*** (0.0788)	−0.1564 (0.0962)	0.5844*** (0.0842)	−0.2924*** (0.0710)	0.8231*** (0.0522)	−0.2023* (0.1054)
External debt (% of GDP)	0.0012*** (0.0003)	0.0010*** (0.0003)	0.0011*** (0.0004)	0.0010*** (0.0003)	0.0013*** (0.0003)	0.0010*** (0.0003)
Household consumption per capita	0.0001 (0.0003)	0.0004 (0.0003)	0.0003 (0.0003)	0.0006** (0.0003)	0.0007 (0.0004)	0.0005 (0.0005)
Out-of-pocket payments	−0.0558*** (0.0063)	−0.0695*** (0.0080)	−0.0614*** (0.0071)	−0.0859*** (0.0071)	−0.0751*** (0.0085)	−0.1036*** (0.0083)
Health shocks (Ebola and COVID)	−1.1642*** (0.2489)	−0.5060** (0.2531)	−0.8769*** (0.3404)	−0.3850 (0.2776)	−0.9356*** (0.3626)	−0.1923 (0.3399)
bwidth_req	0.82	0.71	0.81	0.76	0.82	0.83
bwidth_max	2.02	1.90	4.64	4.72	1.65	1.81
Observations	868	849	868	849	868	849

Robust standard errors in parentheses, ****P* < .01, ***P* < .05, **P* < .1.

**Table A3.** SIVQR estimates of remittances and governance indicators on public health expenditure.

	Public health	Public health	Public health	Public health	Public health	Public health	Public health	Public health
Variables	0.5	0.5	0.5	0.5	0.5	0.5	0.5	0.5
Tax (% of GDP)	0.0013*** (0.0005)	0.0018*** (0.0004)	0.0011*** (0.0004)	0.0014*** (0.0004)	0.0011*** (0.0004)	0.0010*** (0.0004)	0.0013*** (0.0004)	0.0013*** (0.0005)
Health aid	0.4807*** (0.0898)	0.7384*** (0.0396)	0.2669*** (0.0682)	0.2849*** (0.0769)	0.2768*** (0.0797)	0.2167*** (0.0716)	0.1459** (0.0718)	−0.1280 (0.0937)
External debt (% of GDP)	0.0008*** (0.0003)	0.0011*** (0.0003)	−0.0001 (0.0003)	−0.0000 (0.0003)	0.0003 (0.0003)	0.0002 (0.0003)	0.0004 (0.0003)	0.0001 (0.0003)
Household consumption per capita	−0.0001 (0.0003)	−0.0002 (0.0002)	−0.0005* (0.0003)	−0.0007*	−0.0007* (0.0004)	−0.0008** (0.0003)	−0.0007** (0.0003)	−0.0013*** (0.0004)
Remittances	−0.0001 (0.0012)	−0.0021** (0.0010)	0.0027** (0.0012)	0.0038*** (0.0013)	0.0031** (0.0014)	0.0045*** (0.0012)	0.0046*** (0.0012)	0.0101*** (0.0016)
Remittances*gov		−0.0032 (0.0030)						
Governance index (gov)		5.4547*** (1.6459)						
Remittance*va			−0.0000 (0.0000)					
Voices and accountability subindex (va)			0.0047*** (0.0013)					
Remittances*rq				−0.0000* (0.0000)				
Regulatory quality subindex (rq)				0.0037*** (0.0013)				
Remittance*rol					−0.0000 (0.0000)			
Rule of law subindex (rol)					0.0036*** (0.0014)			
Remittance*coc						−0.0000*** (0.0000)		
Control of corruption subindex (coc)						0.0047*** (0.0012)		
Remittance*ge							−0.0000*** (0.0000)	
Government effectiveness subindex (ge)							0.0053*** (0.0012)	
Remittance*psvt								−0.0000*** (0.0000)
Political stability, voilence, and terrorism subindex (psvt)								0.0103*** (0.0015)
bwidth_req	0.85	0.64	0.80	0.89	0.85	0.84	0.88	0.96
bwidth_max	4.26	3.65	4.49	4.96	4.78	4.69	4.92	5.41
Observations	1041	906	1041	1041	1041	1041	1041	1014

Robust standard errors in parentheses, ****P* < .01, ***P* < .05, **P* < .1.
